# Benign Intracranial Hypertension: A Diagnostic Dilemma

**DOI:** 10.1155/2012/814696

**Published:** 2012-08-09

**Authors:** Gary Y. Shaw, Stephanie K. Million

**Affiliations:** Kansas City University of Medicine and Biomedical Sciences, Kansas City, MO 64086, USA

## Abstract

Benign intracranial hypertension (BIH) (also known as pseudotumor cerebri and empty sella syndrome) remains a diagnostic challenge to most physicians. The modified Dandy criteria consist of, the classic findings of headache, pulsatile tinnitus, papilledema, and elevated cerebrospinal fluid (CSF) pressure, however, these are rarely collectively present in any one patient. Furthermore, these findings can wax and wane over time. Due to the nature of this disease, both signs and symptoms may be intermittent, making definitive diagnosis difficult. Newer imaging studies, particularly the magnetic resonance venogram (MRV) along with a constellation of correlative findings and associated diseases have given new impetus in the diagnosis, treatment, and pathophysiology of this disease. This has led the authors to offer modifications to the classic Dandy criteria. This report presents three representative cases of BIH highlighting many of the newer advances in both diagnosis and treatment of this perplexing disorder.

## 1. Introduction

In 1937, Dandy presented a report of 22 patients with elevated intracranial pressure not attributed to brain tumors. All of these patients presented with headaches, most also complained of blurred vision, dizziness, vomiting, and drowsiness. The author made mention of many other signs and symptoms that were experienced by these patients including buzzing in the ears, funduscopic abnormalities, stumbling gate, episodic numbness, and drowsiness, to name a few. In this document Dandy explains that the elevations in pressure seem to come and go over time, and are rarely constant. From this report arose the original Dandy criteria for the diagnosis of benign intracranial hypertension. The original criteria was modified by Smith in 1985, and is currently known as the revised Dandy criteria (1–8), which has been uniformly adopted as a diagnostic paradigm for BIH. The current Dandy criteria are: (1) signs and symptoms of increased intracranial pressure; (2) no other neurological abnormalities or impaired level of consciousness (with the exception of CN VI palsy); (3) elevated intracranial pressure (ICP) w/normal CSF composition; (4) a computed tomography (CT) scan which shows no etiology for increased ICP (the original Dandy criteria required ventriculography); (5) no other cause for intracranial hypertension found [1, 2]. These modified criteria fail to mention that the elevation in pressure and the symptoms may wax and wane or give clear examples of the wide array of symptoms that this disorder may present with. Furthermore, newer imaging studies, particularly the Magnetic Resonance Venogram (MRV) and updated treatment methods have given new impetus in the study of this disease. In light of these developments the authors feel that further modifications to the Dandy criteria are in order. This paper presents three representative examples of BIH highlighting many of the newer advances in both diagnosis and treatment of this perplexing disorder and offers salient modifications to the traditional Dandy criteria.

## 2. Patient Number 1

**Figure 1:**
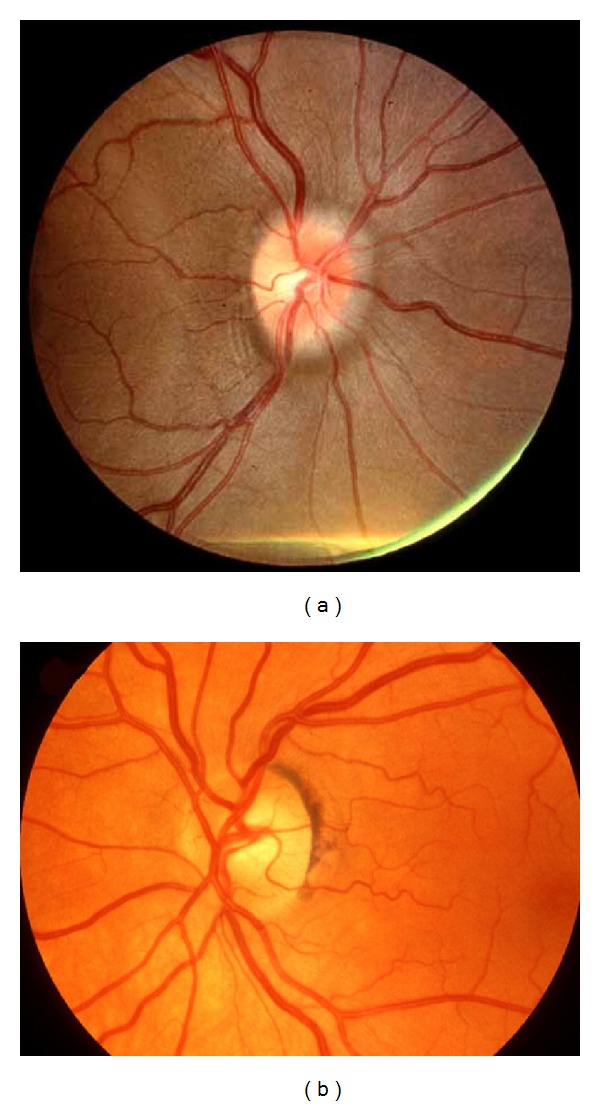
(a) Grade I papilledema [3]. (b) Resolution of papilledema [4].

A 26-year-old, mildly obese Caucasian female presented with a 6-month history of pulsatile tinnitus, right maxillary pressure and pain, and blurred vision in her periphery bilaterally followed by intense, debilitating, daily headaches which extended to her frontal areas bilaterally. The patient denied any trauma associated with the onset of her symptoms. She had been seen by two different physicians recently for this problem; and treated with a course of antibiotics and a pressure equalization (PE) tube was placed in her right ear. Neither treatment aided in the relief of her symptoms. The PE tube appeared to be in good position and patent at the time of her visit. Compression of her right jugular vein led to cessation of the pulsatile tinnitus, suggesting a vascular origin. The remainder of the physical exam was normal, with the exception of mild optic disc blurring bilaterally (Figure 1(a)). Lab testing was found to be negative for rheumatoid factor, C-reactive protein, and ANA, and her erythrocyte sedimentation rate was within normal limits. Review of her head CT scan revealed no intracranial pathology, benign sinuses, and a normal appearing sella turcica. The patient was then referred to a neurologist for further workup.

Over the next 5 months, the patient underwent an extensive evaluation in the neurology clinic and a battery of tests, including a magnetic resonance angiography (MRA) of the venous flow of her head, and the Circle of Willis, magnetic resonance imaging (MRI) of the cervical spine and brain, a lumbar puncture, two magnetic resonance venograms (MRV) of her head, coagulopathy studies, an echocardiogram, and a sleep study. The patient was started on topiramate 50 mg bid for treatment of her headaches. The MRA and MRV both showed signal voids in her transverse and sagittal sinuses, with diminished signal intensity from the left sigmoid and transverse sinuses when compared to the right. Neither study was able to determine whether these findings reflected disease, nondominance, artifact, or a combination of all three. Coagulation studies were positive for the heterozygous presence of the Factor V Leiden (R506Q) mutation, and one copy of the methylenetetrahydrofolate reductase gene mutation A1298C, as well as a slight increase in antithrombin III activity of 129% (normal range 80–120%). Echocardiogram revealed the presence of a right- to- left shunt at rest, consistent with a patent foramen ovale. Polysomnography resulted in the diagnosis of organic sleep apnea and restless leg syndrome. Her lumbar puncture had an opening pressure of 150 mmH20 (normal range 70–180 mmHg) and CSF analysis found no abnormalities. After the lumbar puncture, the pulsatile tinnitus completely resolved. MRV was repeated two weeks after the lumbar puncture and found to be normal without signal voids. This corresponded with the patients clinical assessment of feeling much better.

At 6-months followup the patient continued to do well and her only complaint was mild headaches around the time of menstruation; the topirimate seemed to be working to eliminate her daily headaches, the pulsatile tinnitus remained absent, funduscopic evaluation of her optic discs demonstrated partial resolution of the previously seen papillary edema (Figure 1(b)).

## 3. Patient Number 2

Patient #2, a 43-year-old caucasian female presented with a 13-year history of pulsatile tinnitus which had been getting progressively worse over time. She had been diagnosed as having atypical migraines, seizures, and chronic rhinitis. The patient had undergone numerous workups for these complaints over the years, but the etiology and treatment had yet to be determined. She claimed that the only time that she was without these debilitating symptoms was many years ago when a physician had given her a low dose corticosteroid. She explained that within two weeks of taking the medication her symptoms had completely resolved, however they returned within a few months after the medication had been discontinued and worsened since that time. Her past medical history included a microcytic anemia, hypertension, depression, anxiety, fibromyalgia, sleep apnea, and hypothyroidism, visual changes with floaters, and memory loss. Fundoscopic evaluation demonstrated optic disc swelling. The results of diagnostic tests included a slightly elevated opening pressure on lumbar puncture, an optical CT scan revealing optic disc swelling, and an MRA which was essentially normal. A recent noncontrast brain MRI revealed her pituitary gland was in the upper limits of normal as well as 2 small nonenhancing foci in the left centrum semiovale region which appeared to be chronic. Comparison to other studies was recommended due to these findings. Careful evaluation of the ear drum was completed and no pulsation or bluish discoloration of the tympanic membrane was seen. The patient stated that the tinnitus in her right ear was relieved when slight pressure was applied just beneath her ear.

**Figure 2:**
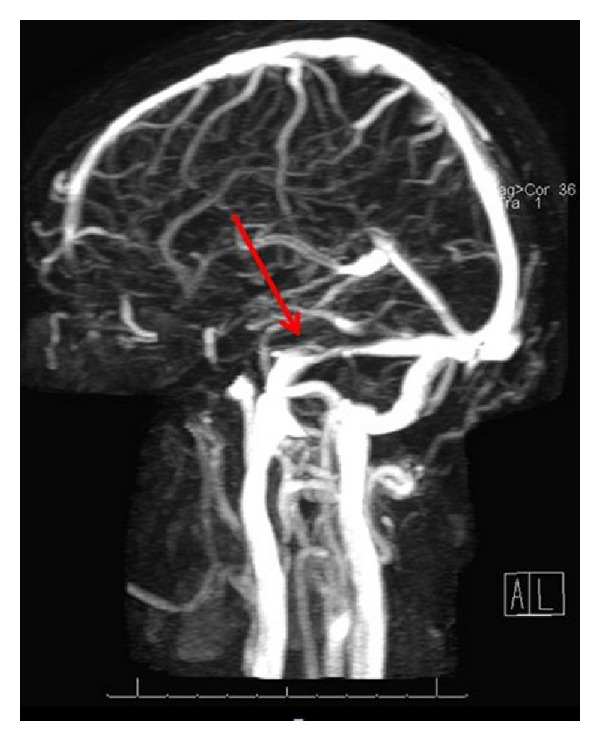
MRV showing narrowing of the transverse venous sinuses; most likely related to benign intracranial hypertension [5].

Since many of her complaints were suggestive of intracranial hypertension, an MRV was performed to look for defects in dural flow, and irregularities with the jugular bulb. The MRV reported filling defects in the superior sagittal sinus and right transverse sinus and right sigmoid sinus (Figure 2). These findings are most often observed secondary to intraluminal thrombus and are consistent with partial thrombosis or recanalization of a dural venous sinus thrombus. Prominence of the cortical draining veins was also found and noted to be most likely caused by partial obstruction due to the filling defects. The report also noted evidence of mastoiditis and suggested this as a likely trigger for thrombus formation. A repeat MRI of the brain was also performed, but revealed no evidence of abnormal enhancing masses, acute infarction, or extra-axial fluid collection. The paranasal sinuses were reported as normal, with some fluid in the inferior portion of the right mastoid air cells, consistent with mastoiditis. After antibiotic therapy, a CT was obtained showing resolution of the mastoid disease. The diagnosis of pseudotumor cerebri was made based on this patient's clinical features and the MRV scans.

She was started on 500 mg of acetazolamide bid. A 6-weeks followup revealed significant resolution of her symptoms. At one year she continued to do well.

## 4. Patient Number 3

**Figure 3:**
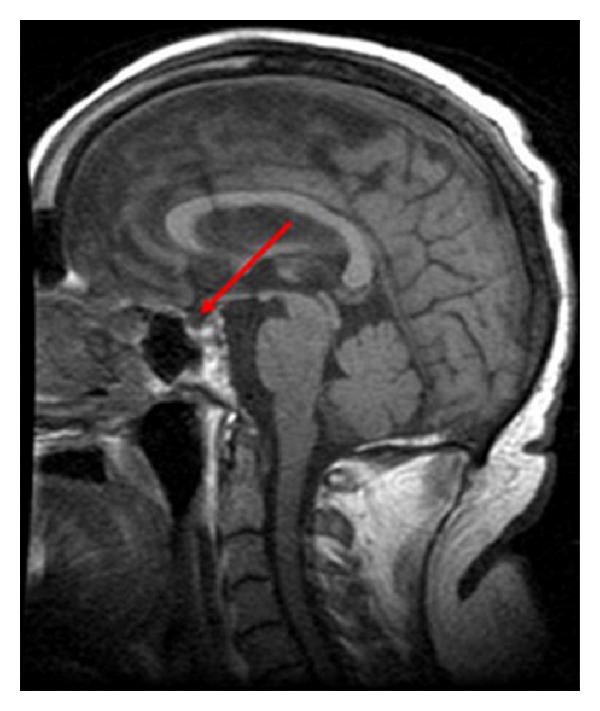
Empty sella on MRI [6].

A 44-year-old African American female presented with complaints of a burning sensation in her tongue for the past 6 months, temporal type headaches, and pulsatile tinnitus. Her medical history included Bell's Palsy, chronic postnasal drip, chronic acid reflux, HTN, numbness and tingling in her right hand, and difficulties sleeping. She had recent seen an ophthalmologist for floaters, colorblindness, and worsening vision. A recent MRI reported that pituitary tissue was not visible within a large sella occupying the posterior aspect of the sphenoid sinus (Figure 3). The study stated that there were flow voids present in the internal carotid and basilar arteries.

Our recommendations included an LP to rule out elevations in intracranial pressure, a sleep study, neurology consultation, and lab testing including TSH, erythrocyte sedimentation rate, and a collagen vascular panel to rule out autoimmune processes.

Upon followup, her lab values were found to be within normal limits, with the exception of hypercholesterolemia, the results of her sleep study were consistent with sleep disordered breathing, the LP was normal, and the neurology consult resulted in the performance of multiple imaging studies. The neurologist found these studies to be necessary as the patient's speech was somewhat dysarthric during the interview and the patient was noted to have slight movement of the left corner of her lips when she blinked. An MRI of the brain reconfirmed the finding of an empty sella, an MRA of the Circle of Willis found no neurovascular abnormalities. The diagnosis of BIH was made based on her clinical presentation and the finding of empty sella. She was started on a carbonic anhydrase inhibitor (acetazolamide) and reported resolution of her symptoms a few weeks after starting the medication.

## 5. Discussion

Three female patients all suffering from pulsatile tinnitus without otologic cause were presented above. After an extensive workup, these patients were found to have many co-morbidities known to be associated with BIH. These include: Factor V Leiden mutation, a patient foramen ovale, sleep apnea, obesity, burning tongue syndrome, neuropathies, anemia, memory loss, disorders in vision, and headaches [7–17]. The medical conditions shared by all three patients were pulstile tinnitus, headaches, sleep apnea, and ophthalmological dysfunctions; which are all consistent findings in benign intracranial hypertension (BIH). In all of these cases an exact cause for pulsatile tinnitus could not be found. The first patient's tinnitus resolved following a lumbar puncture and carbonic anhydrase therapy, thus suggesting that the cause of her presenting complaints was intracranial hypertension. The second patient has a history of abnormally high opening pressure on LP and the third patient was found to have an empty sella on MRI. None of these patients met the diagnostic standards set forth by the current Dandy Criteria; however they all had comorbidities that raised the authors' suspicions of BIH and were all relieved of their symptoms after treatment for elevated intracranial pressure. These cases highlight the challenges in diagnosing and treating patients with BIH and probable relationships between some of the comorbid conditions that, when present with pulsatile tinnitus and an abnormal MRV, should elevate a practitioner's suspicions of BIH and warrant further workup for this condition.

BIH is associated with many medical conditions, including: vitamin deficiencies and excesses, autoimmune diseases, coagulopathies, sleep apnea, obesity, and iatrogenic causes [7–12, 14, 15, 17]. The visual changes and headaches found in patients with BIH have been described in numerous studies and their pathophysiology seems to be well understood [13, 18]. The compression of cranial nerves and intracranial vasculature caused by increased ICP results in a sensation of fullness in the head or cephalgia, while changes in vision and ultimately the complete loss of vision are due to pressure on the optic nerve and papilledema caused by the obstruction of axonal transport at the level of the optic disc.

Pulsatile tinnitus has also been well described. According to Sismanis, the three most common disorders associated with pulsatile tinnitus are BIH, atherosclerotic carotid artery disease, and Glomus tumor [7]. It has been stated that these 3 conditions are the cause of pulsatile tinnitus in 75% of cases, with BIH being the most common cause [7, 10, 19]. Pulsatile tinnitus occurs in approximately 60% of patients with BIH [7, 10, 11, 18–26]. It has also been noted that in a large percentage of patients presenting with pulsatile tinnitus, a definitive cause cannot be found [7, 10, 19–23]. In one study the cause of pulsatile tinnitus was unidentifiable in 27 out of 84 patients (32%), and of these 27 patients pulsatile tinnitus was the presenting symptom in 21 (78%) of them [22].

The pathophysiology of pulsatile tinnitus is believed to be the result of arterial CSF pulsations being transmitted to the compressible medial aspects of the dural venous sinuses resulting in the periodic compression of their walls and luminal narrowing with the conversion of the normal laminar blood flow into turbulent flow [10, 19]. It has been hypothesized that tinnitus and nausea seen in patients with BIH are due to compression of the vestibulocochlear nerve from the increased CSF pressure, which can be explained by the fact that the vestibulocochlear nerve is ensheathed by meningeal coverings that enclose a space in a fashion similar to that of the optic nerve, which make it vulnerable to compression which can cause visual dysfunction [27]. The three patients discussed in this document presented with pulsatile tinnitus, recurrent headaches, and visual disturbances, all of which can be explained by an increase in intracranial pressure using this model.

Many of the other conditions suffered by these patients have not been nearly as well documented. The following information highlights some of these conditions and illustrates their relationship with BIH.

It is estimated that the incidence of BIH ranges from 1-2 per 100,000 persons and that the highest incidence is seen in obese women between the ages of 15–44 [8, 10, 15, 23, 25, 28–31]. Safavi-Abbasi et al. stated that over 80% of patients with BIH are overweight women [30]. With the obesity epidemic rising all over the world, one can be certain that a rise in the incidence of BIH will also occur. A recent study found that of 56 patients with pulsatile tinnitus who were diagnosed with BIH, 37 were obese females [25]. As BIH predominantly affects obese females of child-bearing age (20–44 years), recent research has focused on finding an explanation for these associations. It has been hypothesized that obesity causes an increase in intra-abdominal pressure and venous pressure, and that diaphragmatic elevation in obese patients leads to an increased pleural pressure that results in decreased venous return from the brain to the heart which raises ICP [8, 15, 23, 27, 29–31]. Regarding females of child-bearing age, one plausible explanation is that of an endocrinological dysfunction [23]. Increased amounts of adipose tissue acts as an endocrine organ, releasing hormones such as ghrelin and lecithin, and produces increased levels of estrogen via the aromatization of androstenedione. This can lead to a physiologically abnormal amounts of these hormones in a person's body which may contribute to the development of BIH [8, 23, 29]. One study found that the concentration of estrogen in the CSF from young obese women with pseudotumor cerebri was much greater than found in normal subjects, and that treatment with an 800 calorie/day diet and dexamethasone in obese patients with the disease showed a marked improvement in their clinical outcome [8]. The fact that two of our patients were obese females of child-bearing age, and that the first patient complained of mild headaches associated with hormonal changes in her menstrual cycle further supports the diagnosis of BIH.

It is widely accepted that patients with obstructive sleep apnea (OSA) undergo episodes of hypoxia and hypercapnea which can influence cerebrovascular hemodynamics by increasing cerebral vasodilation which can lead to elevated ICP [18, 26, 30]. Studies have shown that patients with OSA often have normal CSF pressures during the day, but elevated pressures upon awakening and during episodes of apnea [11, 18, 30]. Jennum and Borgesen monitored ICP during sleep in a group of patients with diagnosed OSA and found that all of the subjects developed ICP elevations that were synchronously associated with periods of apnea and that none of these patients had elevated ICP while awake [11]. The study described an initial decrease in ICP during apnea, followed by a slow increase and then a steep increase in intracranial pressures. ICP increases further during sleep, especially during stages N2, N3, and REM in association with apnic episodes. The authors felt the slow increase in ICP was associated with hypercapnia and hypoxia, and indicative of carbon dioxide retention, with the steep increase at the end of the episode being related to an increase in intra-arterial pressure and central venous pressure. These elevations usually increase sympathetic output and total peripheral resistance [11]. They hypothesized that repetitive ICP elevations and variations in cerebral blood flow could predispose patients to an overall increase in ICP, potentially causing cephalgia and cognitive impairment seen with OSA [11, 18]. Other investigators have found that in patients with OSA, intermittent elevations in CSF pressures may cause papilledema and felt that these patients should be considered to have a form of pseudotumor cerebri even though they do not meet the usual diagnostic criteria due to normal measurements of their daytime CSF pressure [18]. All three of our patients were diagnosed with OSA, and the authors believe that a normal opening pressure during daytime lumbar puncture may not be reflective of their CSF pressure while asleep and should not exclude them from being diagnosed with BIH.

A possible relationship between OSA, platelet dysfunction, papilledema, and patent foramen ovale (PFO) is suggested due to the alterations in cerebral and intrathoracic pressure and alterations in blood flow that accompany OSA. It is well accepted that patients with OSA have an increased risk of cardiovascular morbidity; however, the exact etiological mechanism remains elusive. It has been noted that ICP elevations should increase sympathetic tone, which could alter platelet aggregability secondary to increased concentrations of epinephrine and norephinephrine [12, 18]. One study found that treatment with CPAP decreases platelet aggregability, however the exact mechanism of this action is unclear [12]. The increase in intrathoracic pressure seen during apneic episodes would likely have an effect on arterial pressures, total peripheral resistance (TPR), and central hemodynamics. It seems logical that patients with OSA would develop higher BP and possible right heart dysfunction secondary to the increased load on their heart. Shanoudy et al. found an increased prevalence of PFO in subjects with OSA [11, 32], and Beelke et al. [33] documented right- to- left shunting in 9 out of 10 patients with PFO during apneic episodes lasting longer than 17 seconds [11, 31]. These studies do not suggest that OSA is caused by the existence of a PFO, but they do suggest that in subjects with a PFO and OSA, interarterial shunting leads to an increased number of desaturations when compared to patients without a PFO [11]. The finding of a PFO in our first patient does not directly relate to her diagnosis with BIH, however it does lead to questions about the treatment of PFO in patients with OSA.

Many disorders of coagulability, such as factor V Leiden mutation, are associated with an increased occurrence of BIH and a heightened risk of venous thrombosis [8, 22, 34]. In normal individuals activated protein C (APC) inactivates coagulation factor V, which slows down the clotting process. In patients with the factor V Leiden mutation, factor V cannot be inactivated normally by APCY, causing an increasing risk of developing abnormal blood clots [17, 35]. It is possible that a reduction in CSF reabsorption secondary to microthrombus formation damaging arachnoid villi can lead to increased intracranial pressure and BIH [34]. It is estimated that the factor V Leiden mutation is found in 5–10% of the general population and in 20–60% of patients with thromboembolic disorders [22, 35]. Our first patient was found to be positive for the factor V Leiden mutation giving further credence to the diagnosis of BIH. None of her radiological studies were able to confirm the presence of a blood clot; however, the possibility remains that her arachnoid villi were damaged by the formation of microthrombi relating to her coagulopathy.

Burning tongue syndrome (BTS; also known as burning mouth syndrome, glossodynia, glossopyrosis, glossalgia, stomatodynia, oral dysesthesia, and stomatopyrosis) was seen in the third patient described above. BTS is observed principally in middle-aged patients and postmenopausal women and is frequently associated with other sensory disorders such as dry mouth or taste alterations, and its pathogenesis remains unknown [36–38]. Research suggests that abnormalities in the nervous system at the level of the chorda tympani and/or glossopharyngeal nerves play a key role in this disorder. Taste sensation of the anterior two-thirds of the tongue is supplied by the chorda tympani while mechanical and thermal sensations are supplied by the lingual nerve; inhibitory influence between the two are thought to maintain a sensory balance in the tongue [37]. Taste sensation conveyed by the chorda tympani inhibits the area of the brain which receives the afferent impulses from the glossopharyngeal and trigeminal nerves—thus intensifying trigeminal sensations of pain, touch, and dry mouth [38]. Any disruption between these two pathways could potentially lead to an imbalance in the sensory perception of the tongue and cause alterations in taste and sensation as seen in patients with BTS. In 2007, Algahtani et al. utilized electrical detection thresholds to monitor taste/tingling detection in patients with BTS, and determined chorda tympani nerve dysfunction to be present in 82% of their subjects, suggesting hypofunction of this nerve may be a sufficient cause of BTS [37]. Our third subject was noted to have movement of her mouth when blinking, which is a sign of a central nervous system dysfunction. It has been suggested that this blink reflex may be due to diminished presynaptic dopaminergic inhibition, which has also been confirmed in patients with burning mouth syndrome (BMS) [38]. Alterations in cranial nerve function are often used as diagnostic tools in the evaluation of cranial pathology. Trigeminal neuralgia and headache have been noted as in patients with elevated CSF pressure and BIH [39, 40]. Elevated CSF pressures affecting cranial nerves may also be etiologic for BTS.

Benign intracranial hypertension is a condition of increased intracranial pressure without an obvious underlying brain pathology and no evidence of venous thrombosis [13, 15, 22, 23, 29, 30]. There are many proposed etiologies for this very serious medical condition; however, the exact pathogenesis is still unclear. While this condition is labeled as “benign,” that is certainly not the case for many patients with the disorder who suffer from disabling headaches and permanent visual loss [28]. The classic presentation of BIH consists of headache, visual disturbances, pulsatile tinnitus (occurring in approximately 60% of patients), and dizziness, which can be incapacitating for some [10, 15, 21, 23, 24, 27, 28, 30, 41]. As is evident from a review of the current literature, BIH can present with a wide variety of symptoms, as described above. When any of these conditions are present in combination with signs of increased intracranial pressure a physician's suspicions should be heightened towards the diagnosis of BIH. Currently, the diagnosis of BIH requires that the patient meet all of the modified Dandy criteria [9, 10, 13, 15, 29, 42].

1. Signs and symptoms of increased intracranial pressure.
2. No other neurological abnormalities or impaired level of consciousness.
3. Elevated CSF opening pressure with normal CSF composition.
4. A neuroimaging study that shows no etiology for increased ICP.
5. No other cause for intracranial hypertension found.

Our cases demonstrate that the criteria laid out by Dandy may be too rigid and do not take into account fluctuations in presentation, associated conditions, recent imaging, and other clinical details. Therefore, it may be necessary to revise these criteria. The authors believe that in patients presenting with a host of symptoms including pulsatile tinnitus, chronic headaches, visual changes, burning tongue, and other crainioneuropathies, BIH should be considered in the differential diagnosis. If sleep apnea, and/or obesity, and coagulopathies are associated than suspicion should be further raised. The evaluation should include a brain MRV if possible. The authors believe modifications to the current guidelines should include treatment for BIH when patients present with signs and symptoms of increased ICP which are present (constantly or intermittently) and cannot be attributed to any other cause and meet 2 or more of the following:

- Patient is of the female gender and obese,
- Diagnosis of OSA,
- Cranial nerve deficits that cannot be attributed to another cause; these include burning tongue syndrome, trigeminal neuralgia, Bell's Palsy and other similar conditions,
- Elevated opening pressure on LP w/normal CSF compositions (not required to make the diagnosis since fluctuations occur),
- The presence of empty sella &/or flattening of the posterior sclera on neuroimaging
- Cerebral venous abnormalities on MRV, especially narrowing of the venous sinuses, with no apparent cause.

Our rationale relies on the findings of the magnetic resonance venogram (MRV). Current guidelines state MRI as being the preferred method of imaging; even though it is less specific than MRV [42]. Recent literature states that magnetic resonance venography (MRV) of patients with BIH often shows narrowing of cerebral venous sinuses, that can be attributed to a variety of causes including artifact and anatomical variation [22, 28, 29, 43]. It has been noted that MRV, which measures signals from the movement of protons in the blood within a range of velocities chosen by the operator, is often difficult to read when determining the significance of signal voids in the lateral sinuses since they often present with a wide variation in anatomy [43]. Recent studies using MRV have found sinovenous stenosis to be a common finding in up to 90% of patients with BIH [9, 15, 28, 30, 43]. One study used auto-triggered elliptic-centric-ordered three-dimensional gadolinium-enhanced MRV to evaluate 29 patients with BIH and found sinus structural abnormalities in 27 of them [28]. Other studies utilized catheters placed into the intracranial venous sinuses or manometry to measure pressure gradients across the sinuses, and found an increase in pressures within the sinuses in a majority of patients with BIH [9, 29, 30, 43]. These findings have led to the current “chicken or egg” debate as to which came first: the BIH or the venous stenosis; unfortunately there is currently no answer to this question [9, 15, 28–30, 43]. The Monroe-Kellie doctrine, which supports the idea that stenosis is the consequence of BIH, states that in the confined intracranial space the vascular compartment will give way to an expanding parenchymal or CSF compartment and that as more accommodation is required, a threshold is exceeded above which the overall intracranial pressure is elevated and the patient becomes symptomatic [9]. Other studies have found that the stenosis can be reversed after the completion of CSF diversion procedures, thus suggesting BIH causes the stenosis [28, 30]. Our first 2 patients were both found to have abnormalities on MRV. In the first patient signal voids were found in the transverse and sagittal sinuses; the second was found to have filling defects in her superior sagittal, right transverse, and right sigmoid sinuses, which were reported to be consistent with thrombosis, however, no clot was detected on MRV or MRA. A normal MRV was documented following lumbar puncture in the first patient, thus supporting the idea that BIH is the cause, rather than the effect, of venous sinus stenosis. Repeat studies on the second patient found no abnormalities which suggest that the filling defects were the result of compression caused by increased intracranial pressure and that the defects can be reversed once the pressure is removed. This suggests that fluctuations in ICP that can occur over time may make diagnosis of BIH via lumbar puncture or radiographic imaging very difficult since BIH may be a waxing and waning condition in some patients. Transient changes in intracranial pressure have been documented in patients whose ICP was monitored continuously in an attempt to clarify the probably association between sleep apnea and BIH.

Therapy is directed at reducing intracranial CSF pressure, management of symptoms and preservation of vision. The traditional treatment of repeated lumbar puncture and surgical shunting have been supplanted by medical therapy and weight loss. Carbonic anhydrase inhibitors such as acetazolamide are considered first line treatment for BIH [42]. Studies have documented acetazolamide's success in the management of symptoms and stabilizing vision in 47–67% of patients [42]. Topiramate is a migraine therapy with CAH inhibition, making it another option. Furosemide and other diuretics cause nonspecific volume decrease throughout the body, and therefore may be considered as adjuvant therapy with a CAH inhibitor. Mannitol is another diuretic that can be considered a treatment option since the primary mechanism of action is decreasing fluid within the CNS. Lumbar puncture and shunting should be reserved for severe or intractable cases. Associated morbidities such as obesity and obstructive sleep apnea should be aggressively treated.

## 6. Conclusion

BIH remains a diagnostic and therapeutic challenge. Recent advancements in imaging and clinical research have necessitated a new approach to this fascinating disease. With increase in clinical impetus, better diagnostic and therapeutic management will hopefully be forthcoming. Utilizing a revised, less rigid, and diagnostic criteria may aid physicians in making the diagnosis of BIH, and permitting optimal treatment of these patients.
